# Duodenal metastasis from male breast cancer: a case report and review of the literature

**DOI:** 10.4076/1752-1947-3-8331

**Published:** 2009-07-16

**Authors:** Alberto Bruno Ferrari, Giuseppe Pulcini, Federico Gheza, Alessandro Vinco, Stefania Manenti, Edoardo Cervi, Vincenzo Villanacci, Giancarlo Cervi

**Affiliations:** 1 Division of Surgery, Hospital of Gardone Val Trompia, Spedali Civili, Brescia 25063, Italy; 2 Second Division of Anatomic Pathology, Spedali Civili, Brescia 25123, Italy; 3 Third Division of Surgery, Spedali Civili, Brescia 25123, Italy

## Abstract

**Introduction:**

Breast cancer is the most frequent type of tumor and the second leading cause of death in women. Metastases are present in nearly 60% of cases at the time of diagnosis with the lymph nodes, skeleton, lungs, brain and liver as the most frequent sites of metastases. Gastrointestinal involvement is rare, present in only 10% of all the cases. There is a very low risk of developing breast cancer in men.

**Case presentation:**

A 68-year-old man, with a past history of ductal breast cancer, presented with duodenal obstruction. Medical treatment was attempted without success, so he underwent surgery with subtotal gastrectomy and resection of the first portion of the duodenum. Histological examination showed a duodenal metastasis originating from the previous carcinoma of the breast. Five months after surgery, the patient is alive and well.

**Conclusion:**

Gastrointestinal metastases should be considered in patients with a past history of breast cancer. Surgical treatment should be performed in patients who are symptomatic and in good general condition. To our knowledge this is the only case of a gastrointestinal metastasis from breast carcinoma in a man.

## Introduction

Breast cancer is the most frequent type of tumor and the second leading cause of death in women. [1]. Metastases are present in nearly 60% of cases at the time of diagnosis. The most frequent sites of metastases are the lymph nodes, skeleton, lungs, brain and liver. Gastrointestinal involvement is rare, and is detected in only 10% of all the cases [2].

There is a very low risk of developing breast cancer in men; in the US there was an expectancy of nearly 2000 new cases of male breast cancer in 2008 [1]. Gastrointestinal metastasis usually derives from lobular breast cancer rather than the much more common cell type of ductal breast cancer. We report an extremely rare case of a 68-year-old man who presented with intestinal obstruction due to a solitary duodenal metastasis from an infiltrating ductal carcinoma of the breast 40 months after breast surgery.

## Case presentation

In December 2007 a 68-year-old man presented to our institution as an emergency case with abdominal pain, intractable vomiting and weight loss. There was no family history of breast cancer or of other tumors; neither was there any sign of exposure to epidemiologic risk factors.

His relevant past history included an infiltrating ductal carcinoma of the left breast for which he underwent a left mastectomy with Halsted procedure in 2004. Prior to the mastectomy, assessment had included a chest X-ray, bone scan and an abdominal ultrasound, all of which were negative. His carcinoembryonic antigen (CEA) and CA 15-3 results were in the normal range. A review of the histology from his mastectomy specimen showed an infiltrating ductal carcinoma of the left breast. The tumor was 36 mm in maximum diameter infiltrating the muscular tissue and ulcerating the skin; there was a distance of 2 mm from the resection margins. Nine out of 22 lymph nodes isolated from the axillary cavity showed evidence of metastases. Both estrogen and progesterone receptors were positive. The expression of human epidermal growth factor receptor 2 (HER 2) protein was positive (2+). However, a single focus of cribriform carcinoma was found in the central lump.

The patient refused chemotherapy and was only treated with hormonal therapy. Forty months after his first operation, symptoms of upper intestinal obstruction appeared.

A CT scan of his head, chest and abdomen did not show any signs of metastases. A gastroscopy revealed a hemorrhagic duodenal ulcer with stenosis. This was treated successfully with proton pump inhibitors (PPI); no biopsies of the ulcer were taken in consideration of the hemorrhagic risk, while biopsy of the gastric antrum showed an helicobacter pylori (HP) negative gastritis. The patient was discharged from the hospital with a scheduled follow-up gastroscopy in one month.

The esophagogastroduodenoscopy (EGD). performed in January showed the healed ulcer with duodenal substenosis; the patient's clinical condition improved and there were no signs of intestinal obstruction. The benign nature of the lesion was hypothesized and it was decided that biopsy was not needed. Further, an endoscopic follow-up was scheduled in three months.

In March 2008, the patient was again admitted to the hospital with the same symptoms. A clinical examination revealed a distended abdomen. The patient underwent a gastroscopy that showed a duodenal stenosis with hemorrhagic ulcer. A barium upper gastrointestinal (GI) study confirmed the diagnoses. An endoscopic dilatation was attempted without success.

**Figure 1:**
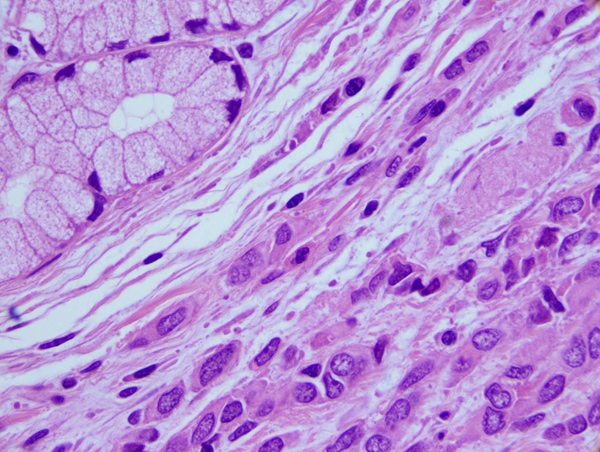
**Metastases of breast carcinoma in the duodenum**. Upper left corner Brunner glands and bottom right neoplastic cells arranged in a "string of beads" of Eosin Ematosillina (100x).

A few days later the patient underwent exploratory laparotomy, subtotal gastrectomy and resection of the first portion of the duodenum. No signs of peritoneal carcinomatosis were detected. On histopatological examination of the gastroduodenectomy, a poorly differentiated carcinoma composed of nests or single strands of atypic cells was identified in the duodenum (Figure 1). Lymph nodes of greater and lesser curves did not show evidence of metastasis. The proximal and distal resection margins were tumor-free.

**Figure 2:**
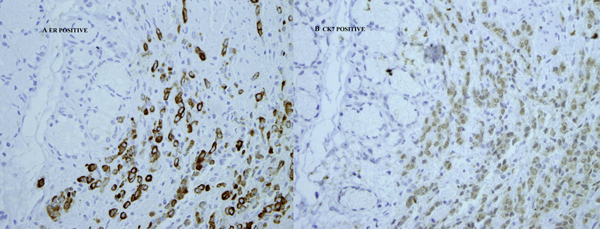
**Neoplastic cells positive for cytokeratin 7 and estrogen receptors**. Upper left corner duodenum bottom right neoplastic cells positive for cytokeratin 7 in frame A and estrogen receptors in frame B (60x).

**Figure 3:**
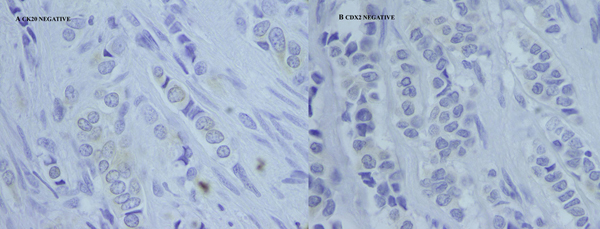
**Neoplastic cells negative for cytokeratin 20 and caudal type homeobox transcription factor 2**. Neoplastic cells negative for cytokeratin 20 in frame A and caudal type homeobox transcription factor 2 in frame B (100x).

Immunohistochemical stains were positive for cytokeratin (CK) 7 and estrogen receptors (ER) (Figure 2), whereas staining with CK20 and CDX2 was negative (Figure 3). On the basis of the histological and immunohistochemical patterns of the tumor, a diagnosis of metastatic breast carcinoma was made.

The patient's recovery after the operation was uneventful. The patient is alive and well and is responding well to the systemic therapy. There have been no signs of recurrence five months after surgery.

## Discussion

There is a very low risk of developing breast cancer in men; in the US there was an expectancy of nearly 2000 new cases of male breast cancer in 2008 [1]. Breast cancer is the second leading cause of gastrointestinal metastasis in women. In clinical practice, a diagnosis of gastrointestinal metastasis from breast cancer is underestimated. Autopsy studies showed that the frequency of gastric metastasis varies between 7.4% and 18%, while a clinical study of gastric involvement with breast cancer described only a 6% frequency during a five-year follow-up of 596 patients [3]-[5].

In McLemore's experience with 12,001 patients with metastatic disease secondary to breast cancer, only 73 had gastrointestinal involvement. Twenty-three of these patients had GI metastases, 32 had carcinomatosis and 18 had GI metastases and carcinomatosis [6].

Clinical diagnosis can be difficult due to nonspecific symptoms like nausea, vomiting, dyspepsia, weight loss and epigastric pain, all of which can be attributable to chemotherapy, radiotherapy or liver metastasis. The mean interval between a diagnosis of breast cancer and presentation with a gastrointestinal metastasis can vary from a few months to several years. Ayantunde et al. describe a mean interval of 6.5 years ranging from 2.8 years to 32.8 years [7]. In Taal's experience the mean interval was four years ranging from 2 to 210 months [8]. In McLemore's series the mean interval between the primary diagnosis of breast cancer and GI metastatic presentation was seven years. In 12 patients (16%) the GI metastases was present at the time of their breast cancer diagnosis [6].

Lobular carcinoma of the breast is less common, representing only 10% to 15% of breast cancer. However, it accounts for 80% of gastrointestinal metastases from breast cancer. Taal et al. described 51 patients with gastric metastases from breast cancer, 70% of which came from infiltrating lobular tumor, while the primary histological type was infiltrating ductal in only 15 cases [8,9]. McLemore states that, compared with the 12% frequency of infiltrating lobular carcinoma in women with primary breast cancer, the prevalence of infiltrating lobular carcinoma in women with GI metastases was significantly increased (54%) [6].

Gastrointestinal metastasis involves the upper gastrointestinal tract more frequently than the large bowel. An endoscopic diagnosis can be difficult due to implantation of the metastatic cells in the submucosa. A biopsy can be negative for tumor cells in over 30% of cases. Imaging techniques like CT scan or ultrasound-guided biopsy can help in the diagnosis.

Immunohistochemistry is the most secure means of making a diagnosis of breast cancer in these patients. Metastases are usually positive for gross cystic disease fluid protein 15 (GCDFP-15), cytokeratin 7, CEA, and estrogen and progesterone receptors; however, they are negative for cytokeratin 20 [10]. The presence of a gastrointestinal metastasis from breast cancer is a sign of a systemic disease which suggests poor survival; in Taal's experience the median survival in patients with a gastric metastasis is 10 months with a 2-year survival of 23% due to some long-term survivors [8].

Late diagnosis of gastrointestinal metastasis is common, and so is the presence of concurrent metastases at the time of the diagnosis. In Taal's series of gastric metastases, most patients (94%) suffered from concurrent metastases, mainly to the skeleton but also to the liver and lungs [8].

Due to its multicentric involvement, systemic therapy with chemotherapy and/or hormonal therapy represent the best adjuvant therapy options. Surgical treatment should be taken into consideration for the treatment of intestinal obstruction or in the rare case of a single metastasis elsewhere. In Taal's series of 51 patients, only six patients underwent surgery, all of them for palliation only, to alleviate severe symptoms of obstruction [8]. In McLemore's study of 23 patients with GI metastases, 12 underwent palliative surgery with a median survival of 44 months compared to the median survival of nine months of patients who were not treated surgically. Surgery did not affect the survival of patients with carcinomatosis [6]. All the data in the literature refers to women. To our knowledge there is no report of a gastrointestinal metastasis in a man.

## Conclusions

In patients with a past history of breast cancer who present with gastrointestinal symptoms, the presence of a metastasis should be taken into consideration.

In a patient in good general condition, with the presence of a single symptomatic metastasis, surgical treatment should be considered strongly, especially when intestinal obstruction symptoms are present. In this patient, surgical treatment, without a histological diagnosis, was undertaken to cure the symptoms.

In this patient, the metastasis appears to have been completely resected. The patient's recovery after his operation was without complication. His obstructive symptoms are no longer present.

To our knowledge this is the first report in the literature of a duodenal metastasis from ductal breast carcinoma in a man.

## Abbreviations

CEA: Carcinoembryonic antigen; CK: Cytokeratin; CT: Computer Tomography; CDX2: Caudal Type Homeobox Transcription Factor 2; ER: Estrogen Receptors; GCDFP-15: Gross Cystic Disease Fluid Protein 15; GI: Gastrointestinal; HER2: Human Epidermal Growth Factor Receptor 2; PPI: Proton Pump Inhibitors.

## Consent

Written informed consent was obtained from the patient for publication of this case report and any accompanying images. A copy of the written consent is available for review by the Editor-in-Chief of this journal.

## Competing interests

The authors declare that they have no competing interests.

## Authors' contributions

ABF is the principal author of the paper, GP conceived the case report, FG and EC helped in collecting the data, VA contributed in writing the introduction and the discussion, VV and SM revised and edited the histopathology description, and GC undertook the final revision before submission. All authors read and approved the final manuscript.
